# Real-world study in severe eosinophilic asthma patients refractory to anti-IL5 biological agents treated with benralizumab in Spain (ORBE study)

**DOI:** 10.1186/s12890-021-01785-z

**Published:** 2021-12-18

**Authors:** Eva Martínez-Moragón, Ismael García-Moguel, Javier Nuevo, Gustavo Resler, ORBE study investigators, Ignacio Antépara, Ebymar Arismendi-Núñez, Francisco Casas-Maldonado, Ignacio Dávila-González, Ismael García-Moguel, Jose Luis Velasco-Garrido, Rocío Díaz-Campos, Carmen Díaz-Donado, Beatriz Gálvez, Jose Luis Izquierdo, Antolín López-Viña, Eva Martínez-Moragón, Cristina Navarro-Soriano, Marta Palop-Cervera, Luis Pérez de Llano, Vicente Plaza, Miguel Ángel Racionero-Casero, Manuel Rial-Prado, Marina Carmen Rodríguez-Hernández, Maria Jesús Rodríguez-Nieto, Miguel Zabaleta

**Affiliations:** 1 Pneumology Service, University Hospital Doctor Peset, Valencia, Spain; 2 Severe Asthma Unit, Allergy Department, Hospital Universitario, 12 de Octubre, Madrid, Spain; 3 AstraZeneca Farmacéutica Spain S.A., Madrid, Spain

**Keywords:** Asthma, Severe asthma, Eosinophilic asthma, Benralizumab, Biological treatment, Early access programme

## Abstract

**Background:**

Benralizumab, a monoclonal antibody targeting the human interleukin-5 (IL-5) receptor (IL-5R), was used before marketing authorisation in Spain in a real world setting as part of an early-access programme (EAP) to treat patients with severe eosinophilic asthma with prior insufficient response or intolerance to anti-IL5 treatment (mepolizumab or reslizumab). The objective of this study is to describe the patient profile candidate for treatment and to assess benralizumab effectiveness.

**Methods:**

This is an observational, retrospective, multicentre study in severe eosinophilic asthma patients refractory to other biological agents targeting the IL-5 pathway. Baseline characteristics included closest data, from the previous 12 months, to benralizumab treatment onset (index date). Patients were followed until the last treatment dosage while EAP was active (March to December 2018). Effectiveness was evaluated versus baseline, in patients who received at least three doses, with asthma control test (ACT), Mini Asthma Quality of Life Questionnaire (MiniAQLQ), annual severe exacerbation rate, oral corticosteroids treatment (OCS) and asthma-related healthcare resources utilization.

**Results:**

Twenty-seven patients treated with benralizumab were included in the analysis. Effectiveness was assessed in 19 patients. Both questionnaires showed clinically meaningful differences, i.e. ACT score ≥ 3 and MiniAQLQ score ≥ 0.5, compared with baseline [mean (SD), 3.3 (6.8) and 1.2 (1.9), respectively]. Patients treated with OCS decreased during follow-up from 88.9% (n = 24/27) at baseline to 78.9% (n = 15/19) and 31.6% (n = 6/19) had an OCS dose reduction ≥ 50%. The difference in annual severe exacerbation rate during follow-up showed a significant reduction vs. baseline (2.12 per patient-year, 95% CI 0.99–3.24, p = 0.002). The differences in annual rate of non-scheduled primary care and specialist visits during follow-up indicated a significant decrease [2.28 per patient-year (95% CI 1.55–3.01; p < 0.001) and 1.47 per patient-year (95% CI 0.65–2.30; p = 0.004), respectively], as well as the difference in annual rate of number of emergency department visits [1.18 per patient-year (95% CI 0.51–1.85; p = 0.007)].

**Conclusions:**

These results suggest that severe eosinophilic asthma patients receiving benralizumab, presented clinically meaningful improvement in asthma control and asthma-related QoL as well as OCS dose reduction. Results also aim to significant reductions in annual severe exacerbation rates, non-scheduled primary care and specialist visits, and emergency department visits rates.

## Background

Asthma is a chronic disease with a significant impact at personal, social and economic level with important implications in healthcare resources utilization involving those inherent in the disease treatment (emergency visits, hospitalisation, medical care, treatment costs, etc.) [1]. The burden of disease increases with increasing severity and lack of control [1, 2].

The prevalence of asthma in adults in Spain is approximately 5% [3]. Between 6 and 10% of all patients develop severe asthma and require long-term treatment with high dose inhaled corticosteroids (ICS) plus long-acting ß2-adrenergic agonists (LABA) and even, oral corticosteroids (OCS) to reach the target of asthma control [4]. Despite these treatments, most of these patients still have poor disease control, persistent limitation of airflow with frequent and severe exacerbations [5, 6].

According to the inflammatory cells present in blood, sputum or bronchial biopsy, there are different types of asthma phenotypes regarding the predominance of eosinophils, neutrophils, both cell types or none of them [7–9]. The current and future therapeutic approaches to asthma should include this stratification of patients according to these phenotyping criteria [10].

Several monoclonal antibodies are currently approved as add-on treatment for severe asthma patients in Spain. Omalizumab, mepolizumab, reslizumab, benralizumab and dupilumab have demonstrated efficacy in randomised clinical trials [11–16].

Benralizumab is a humanised monoclonal antibody (IgG1) which binds specifically to the alpha subunit of the receptor of the IL-5 present in eosinophils and basophils and results in apoptosis of these cells through antibody-dependent cell-mediated cytotoxicity (ADCC) by natural-killer (NK) and other cells [17]. Benralizumab has demonstrated efficacy in reducing severe exacerbations, increasing lung function, improving asthma-control and reducing, or even eliminating oral corticosteroids, regardless of the atopy status [14, 15, 18–21].

Few real-life experience data are available to date because of the recent marketing approval of benralizumab. Many questions about responders’ and non-responders’ characteristics, predictors of response, and residual disease after blocking the IL-5 pathway remain unanswered. Information on patients with partial response or no response to other anti-IL5 biologics and the effect of switching them to benralizumab is important to improve personalised treatment.

The Spanish Medicines Agency (AEMPS) authorised a programme in March 2018 for the prescription of benralizumab prior to commercialisation. This early-access programme (EAP) was based on the evidence of the persistent medical burden and unmet medical need of severe asthma patients and the potential efficacy differences between benralizumab and previously approved monoclonal antibodies in severe eosinophilic asthma.

The main objective of this study was to characterize the patient profile and to evaluate the effectiveness of benralizumab in real world setting as part of the EAP in Spain.

## Methods

### Study design and study population

This observational, retrospective, multicentre study involved 25 sites and included patients treated with at least one benralizumab dose during the EAP conducted in Spain between March 2018 and December 2018.

Written informed consent was obtained from all participants. The study was approved by the AEMPS. Additional approvals were obtained by several different ethics committees in compliance with local and regulatory guidelines.

Adults (18-year-olds or older) with severe eosinophilic asthma receiving high-dose ICS in combination with LABA (with or without another controller) unresponsive to other anti-IL5 biological agents available in Spain (mepolizumab or reslizumab), based on the physician’s judgement were included. Benralizumab treatment initiation was also based on the physician’s criteria according to the definition of severe eosinophilic asthma. Patients received the approved benralizumab dose of 30 mg subcutaneously every 4 weeks for the first three doses and then every 8 weeks thereafter [22]. The presence or absence of a washout period between previous treatment and Benralizumab treatment initiation was at the investigator’s discretion.

### Clinical, analytical, asthma-related resource consumption and lung function variables

A database was compiled from patients’ medical records. Baseline characteristics included closest data to benralizumab treatment onset (index date), and patients were followed-up until the last benralizumab treatment dosage while EAP was active (March to December 2018). Sociodemographic data (sex, age), basic blood test and clinical profile (age at diagnosis of asthma, atopy, asthma-related and non-related comorbidities) were collected at index date.

Benralizumab effectiveness was measured comparing baseline versus follow-up only in those patients who received at least three doses of benralizumab, including asthma control (Asthma Control Test [ACT]) [23], lung function (FEV_1_, FVC), quality of life (MiniAQLQ), annual exacerbation rate, oral corticosteroids use, and asthma-related healthcare resource use (non-scheduled primary care and specialist visits, as well as asthma-related ED visits). Annual exacerbation rate and asthma-related healthcare resources use at baseline were referred to the previous 12 months to index date.

Clinical outcomes data were collected. Severe asthma exacerbations (defined as hospital admission, OCS bursts or OCS dose increasing during ≥ 3 days and emergency department asthma visits) and OCS use were collected for the year prior to and after benralizumab treatment initiation. The severity of exacerbations was defined according to the 2009 ATS/ERS consensus statement [24].

ACT is widely used in Spain [25] and available in electronic medical records for asthma control measure. The ACT has been validated in Spanish [26]. A test score under 20 indicates lack of control and a difference ≥ 3 units is considered as clinically meaningful.

QoL was measured at baseline and after benralizumab treatment with the validated Spanish version of MiniAQLQ, self-administered by patients [27, 28]. The questionnaire evaluates four dimensions (symptoms, limitation of activities, emotional sphere and environmental stimulation) based on 15 questions rated 1 (always, worst) to 7 (never, better quality of life). A test score difference ≥ 0.5 is considered as clinically meaningful.

Asthma-related healthcare resources use was also collected from medical records.

### Statistical analysis

For continuous variables, descriptive statistics (n, mean, and standard deviation [SD]) are provided. For categorical variables, absolute frequency and valid percentages (i.e., excluding missing data) are provided. Patient demographic and clinical characteristics were summarised using descriptive statistics. Exacerbation rates in the year prior to treatment were expressed per patient-year and calculated before and after benralizumab treatment initiation as the number of episodes divided by time of exposure. Background controller treatment was also described and summarised using descriptive statistics (mean, SD, n, and valid percentage). For the comparison of the same measurement at two different times, either paired T-test or Wilcoxon was used, depending on the sample distribution. Statistical significance was set at *p* < *0.05*. The analysis was performed using the IBM SPSS Statistics software, Version 22.0 (IBM Corp. Armonk, NY).

## Results

### Clinical, functional, and laboratory data of patients at baseline

Clinical, functional, and laboratory characteristics of the study population at baseline are shown in Table 1.

**Table 1:** Baseline patient characteristics

Parameter	N = 27
Age (years), mean (SD)	49.8 (12.7)
Women, n (%)	14 (51.9)
BMI, mean (SD)	28.4 (5.9)
Smoking, n (%)	
Non-smoker	19 (70.4)
Former smoker	8 (29.6)
Age at diagnosis (years), mean (SD)^a^	30.2 (12.2)
Time since diagnosis (years), mean (SD)	19.2 (13.8)
Asthma phenotype, n (%)	
Eosinophilic	24 (88.9)
Eosinophilic and atopic	3 (11.1)
Pre-BD FEV_1_, mean (SD)^b^	
mL	1,813.3 (480.8)
%	62.1 (14.6)
Post-BD FEV_1_, mean (SD)^b^	
mL	1,989.3 (819.7)
%	65.7 (20.5)
ACT^c^	
Mean (SD)	14 (6.1)
Controlled asthma (ACT ≥ 20), n (%)	6 (22.2)
miniAQLQ, mean (SD)	3.4 (0.7)
FeNO (ppb), Mean (SD)^d^	76.2 (56.5)
Blood eosinophil count (cells/μL), mean (SD)^a^	371.9 (315.5)
< 300 cells/μL, n (%)	11 (40.7)
≥ 300 cells/μL, n (%)	15 (55.6)
Total IgE (IU/ml), mean (SD)^b^	593.1 (1,054.5)
Asthma-treatment in the previous year, n (%)	
ICS + LABA	27 (100)
OCS	24 (88.9)
LAMA	22 (81.5)
LTRA	17 (63)
ICS	8 (29.6)
Macrolides	6 (22.2)
Theophylline	1 (3.7)
LABA	1 (3.7)
Biologic agent	
Mepolizumab (anti-IL5)	23 (85.2)
Reslizumab (anti-IL5)	2 (7.4)
Omalizumab (anti-IgE) (first line)/Mepolizumab (second line)	1 (3.7)
Omalizumab (first line)/Reslizumab (second line)	1 (3.7)
Oral prednisone dose (mg/day), mean (SD)	20.3 (20.1)
Inhaled budesonide (in combination) dose (μg/ day), Mean (SD)	305 (60.2)
Duration of prior biologic therapy (days), mean (SD)	
Duration of prior mepolizumab therapy^a^	250.8 (167.6)
Duration of prior reslizumab therapy	150.7 (58.4)
Duration of prior omalizumab therapy	77 (107.5)
Time since prior biologic therapy (days), mean (SD)^a^	121.4 (110.7)

*ACT* Asthma Control Test, *AQLQ* Asthma Quality of Life Questionnaire, *BD* bronchodilator, *BMI* body mass index, *FeNO* fractional exhaled nitric oxide, *FEV*_*1*_ forced expiratory volume in 1 s, *ICS* inhaled corticosteroids, *IL-5* Interleukin 5, *IU* international units, *LABA* long acting β2-agonists, *LAMA* long acting muscarinic antagonists, *LTRA* leukotriene receptor antagonists, *μL* microliter, *mL* millilitre, *OCS* oral corticosteroids, *ppb* parts per billion, *SD* standard deviation, *SPT* skin prick test

^a^Data unknown in 1 case (3.7%)

^b^Data unknown in 6 cases (22.2%)

^c^Data unknown in 2 cases (7.4%)

^d^Data unknown in 8 cases (29.6%)

A total of 27 severe asthma patients were evaluated, 88.9% (n = 24) had eosinophilic asthma and 11.1% (n = 3) also had atopic features. Mean (SD) time since diagnosis was 19.2 (13.8) years. At baseline, mean (SD) blood eosinophil count was 371.9 (315.5) cells/μL and 55.6% (n = 15) had ≥ 300 cells/μL; mean (SD) ACT score was 14 (6.1) with a total of 21 patients (77.8%) with uncontrolled asthma (ACT score < 20).

All patients were treated with high-dose ICS plus LABA and 24 (88.9%) had been treated with OCS as maintenance treatment prior to benralizumab initiation. Oral prednisone (equivalent) mean (SD) dose was 20.3 (20.1) mg/day. All patients had insufficient response or were intolerant to prior treatment with anti-IL5 or anti-IgE treatment: 24 (88.9%) had been previously treated with mepolizumab, 3 (11.1%) with reslizumab and 2 (7.4%) even received omalizumab before the anti-IL5 treatments.

Most of the patients had ≥ 1 asthma-related comorbidity (74.1%; n = 20) (Table 2). The most frequent were allergic rhinitis (44.4%), nasal polyps (40.7%), gastroesophageal reflux disease (40.7%), and chronic rhinosinusitis (37%) (Table 2).

**Table 2:** Baseline patient comorbidities

Parameter	N = 27
**Asthma-related comorbidities**, mean (SD)	2 (1.5)
Number of asthma-related comorbidities, n (%)	
0	7 (25.9)
1	1 (3.7)
≥ 2	19 (70.4)
Asthma-related comorbidities (frequency > 5%), n (%)	
Allergic rhinitis	12 (44.4)
Nasal polyps	11 (40.7)
Gastroesophageal reflux disease	11 (40.7)
Chronic rhinosinusitis	10 (37)
Allergic conjunctivitis	5 (18.5)
Atopic dermatitis	4 (14.8)
**Other relevant comorbidities** (frequency > 5%), n (%)	
Depression and anxiety	10 (37)
Obstructive sleep apnoea	8 (29.6)
Other diseases	8 (29.6)
Osteoporosis	5 (18.5)
Cardiovascular disease	3 (11.1)
Diabetes mellitus	2 (7.4)

*SD* standard deviation

### Clinical variables assessed after benralizumab treatment initiation

Of the 27 patients evaluated at baseline, 70.4% (n = 19) received at least three doses of benralizumab in the EAP and were evaluated at follow-up. 11.1% (n = 3) received two doses and 18.5% (n = 5) received a single dose. The mean (SD) time between the first and the last dosage in those patients was 5 (2.1) months. Of these 19 patients, 89.5% (n = 17) continued treatment with benralizumab after the EAP had been completed and following the approval and marketing authorisation of benralizumab in Spain. In contrast, two patients (10.5%) discontinued the treatment with benralizumab after the EAP, in one case because worsening of the symptoms, and, in the other case because occurrence of AEs. This information was not available in the case of the patients who received less than three doses of benralizumab in the EAP, according to the study protocol.

#### Asthma control and quality of life

Mean (SD) blood eosinophil counts decreased from 490 (353.9) at baseline to 0.8 (2.8) cells/μl after treatment (p = 0.002) (Table 3).

**Table 3:** Asthma control and QoL at baseline and after benralizumab treatment initiation

Variables	Baseline	After treatment initiation
**Lung function**		
Pre-BD FEV_1_ (mL), N = 10		
Mean (SD)	1,827 (505.9)	1,982 (459.9)
Difference, mean (SD)	155 (430.4)	
p-value*	0.284	
Pre-BD FEV_1_ (%), N = 10		
Mean (SD)	63 (18.8)	68.9 (20.7)
Difference, mean (SD)	5.8 (17.3)	
p-value*	0.314	
Pre-BD FEV_1_/FVC (%), N = 10		
Mean (SD)	59.8 (9.9)	56.2 (24.2)
Difference, mean (SD)	− 3.6 (20)	
p-value*	0.441	
Post-BD FEV_1_ (mL), N = 9		
Mean (SD)	2,093.3 (995.8)	2,350 (1,062.9)
Difference, mean (SD)	256.7 (400.6)	
p-value*	0.091	
Post-BD FEV_1_ (%), N = 9		
Mean (SD)	66.2 (22.1)	73.5 (24.3)
Difference, mean (SD)	7.3 (13.6)	
p-value*	0.147	
Post-BD FEV_1_/FVC (%), N = 9		
Mean (SD)	65.2 (11.8)	67.4 (12.5)
Difference, mean (SD)	2.2 (5.8)	
p-value*	0.291	
** Blood eosinophil count **(cells/μL), N = 13		
Mean (SD)	490 (353.9)	0.8 (2.8)
Difference, mean (SD)	− 489.2 (354.4)	
p-value*	0.002	
**OCS**	N = 27	N = 19
OCS-dependent, n (%)	24 (88.9)	15 (78.9)
OCS dose reduction ≥ 50%, n (%)	6 (31.6)^a^	
Oral prednisone (mg/day), N = 15		
Mean (SD)	15.1 (15.8)	21.8 (18.6)
Difference, mean (SD)	6.7 (2.8)	
p-value*	0.144	
**ACT**, N = 15		
Mean (SD)	14.8 (6.8)	18.1 (6.3)
Difference, mean (SD)	3.3 (6.8)	
p-value*	0.079	
Clinically meaningful difference (≥ 3), n (%)	9 (60)	
** miniAQLQ**, N = 5		
Mean (SD)	3.6 (0.8)	4.7 (1.5)
Difference, mean (SD)	1.2 (1.9)	
p-value*	0.236	
Clinically meaningful difference (≥ 0.5), n(%)	2 (40)	

*ACT* Asthma Control Test, *AQLQ* Asthma Quality of Life Questionnaire, *BD* bronchodilator, *FEV*_*1*_ forced expiratory volume in 1 s, *FVC* forced vitality capacity, *μL* microliter, *mL* millilitre, *OCS* oral corticosteroids, *SD* standard deviation

*Paired T-test

^a^Data unknown in 6 cases after benralizumab treatment (31.6%)

**Fig. 1:**
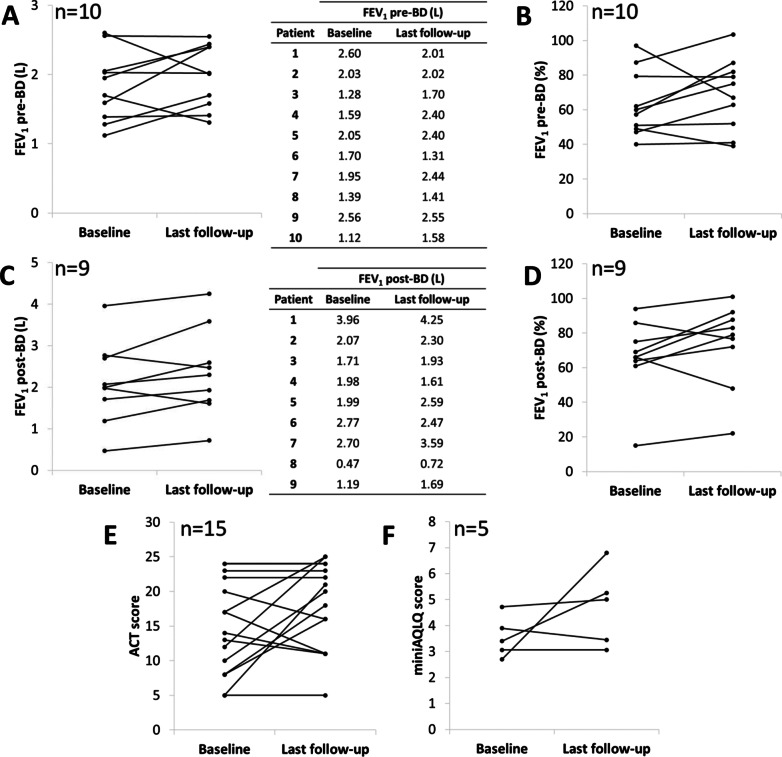
Effect of benralizumab on FEV_1_ pre-BD-L (**A**), FEV_1_ pre-BD-% (**B**), FEV_1_ post-BD-L (**C**), FEV_1_ post-BD-% (**D**), ACT score (**E**), and miniAQLQ score (**F**). *ACT* Asthma Control Test, *AQLQ* Asthma Quality of Life Questionnaire, *BD* bronchodilator, *FEV*_*1*_ forced expiratory volume in 1 s

According to data from the 19 patients that received at least the first three doses, pre-bronchodilator (pre-BD) and post-bronchodilator (post-BD) lung function showed no apparent difference after benralizumab treatment initiation compared with baseline, as showed in Table 3 and Fig. 1A–D. However, 9 (60%) out of 15 patients had a clinically meaningful increase in FEV_1_ of 230 mL.

Patients showed improvement in asthma control based on the ACT score after benralizumab treatment compared with baseline [14.8 (6.8) vs. 18.1 (6.3); p = 0.079] (Table 3 and Fig. 1E). The point estimate difference between the last monitoring value and baseline was 3.3 (6.8), which is numerically larger than the minimal clinically important difference (MCID) (increase of ≥ 3 units). Additionally, 60% (n = 9) of the patients with ACT score recorded at baseline and after treatment initiation (n = 15) achieved a clinically significant response (ACT score difference ≥ 3).

A significant reduction in the proportion of patients (88.9% [n = 24]) receiving OCS at baseline vs. 78.9% (n = 15) after treatment initiation with benralizumab was observed and 31.6% (n = 6) had an overall OCS dose reduction ≥ 50%.

Regarding QoL, although not numerical difference (p = 0.236), a MCID (score difference ≥ 0.5) in miniAQLQ of 1.2 (1.9) was observed vs. baseline (Table 3 and Fig. 1F).

#### Exacerbations and asthma-related healthcare resources use

As illustrated in Table 4, a reduction in the annualized exacerbation rate was observed between the year prior to treatment initiation and the time following benralizumab initiation [4.4 (2.9) vs. 1.9 (1.2) respectively]. The difference in the annual severe exacerbation rate was 2.12 (95% CI 0.99–3.24) (i.e., 3.89 before treatment vs. 1.77 after treatment initiation, nominal p = 0.002) (Table 4). A total of 10 patients did not report exacerbations during follow-up after benralizumab treatment initiation (11.1% of patients before treatment vs. 52.6% during follow-up). In contrast, 71.5% (n = 22) of patients before treatment had ≥ 2 exacerbations in the previous year vs. 21.1% (n = 4) after benralizumab treatment initiation.

**Table 4:** Severe exacerbations in the previous year and after treatment initiation with benralizumab

Variables	Previous year (N = 27)	After treatment initiation (N = 19)
**Severe exacerbations**, mean (SD)	4.4 (2.9)	1.9 (1.2)
**Annual rate of severe exacerbations**		
Rate per patient-year	3.89	1.77
Difference	2.12	
CI 95%	0.99–3.24	
p-value*	0.002	
**Number of exacerbations**, n (%)		
0	3 (11.1)	10 (52.6)
1	2 (7.4)	5 (26.3)
2	6 (22.2)	1 (5.3)
≥ 3	16 (59.3)	3 (15.8)
**Category of exacerbation**, n (%)		
OCS Bursts/dose increasing ≥ 3 days	24 (88.9)	8 (42.1)
Hospital admission	5 (18.5)	2 (10.5)
ED visit	9 (33.3)	5 (26.3)
**Annual rate per category of exacerbation**		
OCS bursts/dose increasing ≥ 3 days		
*Rate per patient-year*	3.93	1.46
*Difference*	2.47	
*CI 95%*	1.40–3.54	
p-value*	< 0.001	
Hospital admission requirement		
*Rate per patient-year*	0.37	0.21
*Difference*	0.16	
*CI 95%*	− 0.21–0.53	
p-value*	0.707	
ED visit requirement		
*Rate per patient-year*	1.41	0.52
*Difference*	0.89	
*CI 95%*	0.25–1.53	
p-value*	0.034	

*CI* confidence interval, *CS* corticosteroids, *ED* emergency department, *ICS* inhaled corticosteroids, *OCS* oral corticosteroids, *SD* standard deviation

*Fisher’s exact test

Regarding asthma-related healthcare resources use, most of the patients had ≥ 1 non-scheduled primary care and specialist visits in the year prior to benralizumab treatment (55.6% and 59.3%, respectively) (Table 5). In contrast, after benralizumab treatment initiation, the mean (SD) number of non-scheduled visits to primary care and specialists registered a reduction from 3.7 (3.9) to 1 (0), and from 3.8 (3.3) to 1.6 (0.9), respectively. The observed differences during follow-up in the annual rate of non-scheduled visits to primary care and specialist were 2.28 (95% CI 1.55–3.01; p < 0.001) and 1.47 (95% CI 0.65–2.30; p = 0.004), respectively. Also, the difference in annual rate of asthma-related ED visits was nominally statistically significant: 1.18 (95% CI 0.51–1.85; p = 0.007).

**Table 5:** Asthma-related resources consumption

Variables	Previous year (N = 27)	After treatment initiation (N = 19)
**Asthma-related non-scheduled visits**		
*Primary care*		
Mean (SD)	3.7 (3.9)	1 (0)
Number, n (%)		
0	7 (25.9)	16 (84.2)
≥ 1	15 (55.6)^a^	2 (10.5)^b^
Annual rate per patient-year	2.50	0.22
Difference	2.28	
CI 95%	1.55–3.01	
p-value*	< 0.001	
*Specialists*		
Mean (SD)	3.8 (3.3)	1.6 (0.9)
Number, n (%)		
0	10 (37)	14 (73.7)
≥ 1	16 (59.3)^c^	5 (26.3)
Annual rate per patient-year	2.31	0.83
Difference	1.47	
CI 95%	0.65–2.30	
p-value*	0.004	
**Asthma-related hospital admissions**		
Mean (SD)	2 (1.4)	1 (0)
Number, n (%)		
0	22 (81.5)	17 (89.5)
≥ 1	5 (18.5)	2 (10.5)
Annual rate per patient-year	0.37	0.21
Difference	0.16	
CI 95%	− 0.21 to 0.53	
p-value*	0.707	
**Asthma-related ED visits**		
Mean (SD)	3.8 (4.4)	1 (0)
Number, n (%)		
0	15 (55.6)	14 (73.7)
≥ 1	12 (44.4)	5 (26.3)
Annual rate per patient-year	1.70	0.52
Difference	1.18	
CI 95%	0.51–1.85	
p-value*	0.007	

*CI* confidence interval, *ED* emergency department, *SD* standard deviation

*Fisher’s exact test

^a^Data unknown in 5 cases (18.5%)

^b^Data unknown in 1 case (5.3%)

^c^Data unknown in 1 case (3.7%)

## Discussion

This study suggests that in real world conditions, benralizumab reduces annual severe exacerbations rates, non-scheduled primary care and specialist visits, and emergency department visits, in patients mainly unresponsive to anti IL-5. Additionally, data suggest an important improvement in asthma control and asthma-related QoL (ACT and miniAQLQ, respectively) as well as a reduction in the percentage of patients treated with OCS. Lung function improvement was clinically meaningful (FEV_1_ change = 230 mL; [29]) in 9 out of 15 patients.

Benralizumab is safe and effective in patients with refractory eosinophilic asthma according to several clinical trials. However, even though real-life data may differ from data obtained from pivotal clinical trials due to their broader profile characteristics compared with those enrolled in clinical trials, our results are in line with pivotal benralizumab studies SIROCCO, CALIMA, and ZONDA [14, 15, 18].

Regarding the number of exacerbations in our study, the rate per patient-year dropped by 56.8% (from 4.4 at baseline to 1.9 after at least three benralizumab doses). In the SIROCCO and the CALIMA studies, reductions in exacerbations were 51% and 28% per year, respectively [14, 15]. This greater improvement compared to the pivotal studies is aligned with previous studies in real-life settings and could be related to broader severity degree and characteristics of patients analysed in usual clinical conditions studies [30, 31].

A real-life retrospective study evaluated data from 15 patients with difficult-to-treat, severe eosinophilic asthma treated with benralizumab for up to 6 months in Italy [31]. This study suggested that benralizumab is effective in improving ACT, AQLQ, and lung functional outcomes as well as in reducing the number of exacerbations. Similarly, a cross-sectional study evaluated 42 severe refractory eosinophilic asthma-patients treated with benralizumab for at least 6 months in Spain [30]. This study not only confirmed the efficacy and safety of the treatment in real-life but also showed a rapid initial and persistent improvement during the first 6 months of treatment in lung function, control and quality of life. In these studies, less than half of the enrolled patients had been treated previously with anti-IL5 antibodies (mepolizumab or reslizumab). An additional observational study in Italy showed that the onset of effect of clinical effects of benralizumab on blood eosinophil count, symptom control, lung function and OCS intake (daily intake tapering from 15.58 (8.30) mg/day to 0 mg/day) appear to be detectable as soon as 4 weeks after initiation of treatment. [32]. In this sense, responses may vary between the different anti-IL-5 biological agents, which may be due to differences in target, mode of administration, or dosing interval [33]. More specifically, real-life data showed enhanced responses to benralizumab in patients with severe eosinophilic asthma and chronic rhinosinusitis with nasal polyps (CRSwNP) [34].

Previously published pivotal lung function improvement results in patients with ≥ 300 eosinophils/μL (measured by FEV_1_ difference with placebo) were 0.159 L (p = 0.0006) and 0.116 L (p = 0.0102) in the SIROCCO and CALIMA studies, respectively [14, 15]. Real-world data results suggest that lung function continues to improve at 3 months and also at 6 months of treatment [30].

In real world conditions, anti-IL-5 (mepolizumab, reslizumab), and anti-IL-5R (benralizumab) biologic agents have shown that more than 80% of patients with severe eosinophilic asthma have a favourable long-term response to these treatments, although switches between biologics were frequent (34% of patients switched to other anti-IL5 or anti-IL5R, and 7% made 2 switches) [33, 35]. Most frequent reported reasons for switching were persistent asthma or sinonasal symptoms, including exacerbations, whereas only a small percentage switched because of adverse events [33].

In this analysis, super response was observed in 14% of patients and only 11% of patients were defined as non-responders [33]. Our results showed a numerically increase in the magnitudes of lung function measurements, both in FEV_1_ and FEV_1_/FVC, whereas no apparent difference after benralizumab treatment initiation compared with baseline. Thus, considering the observed improvement in other outcomes of the study, these changes in the lung function magnitudes that we observed in our results, after at least the first three doses of benralizumab, may continue to improve. Our analysis also showed an improving trend in ACT and miniAQLQ score differences between baseline and after at least the first three benralizumab doses, higher than what are considered as clinically meaningful differences (ACT ≥ 3 and miniAQLQ ≥ 0.5). A recent study in real-life conditions observed that a single dose of benralizumab led to a rapid and remarkable improvement in symptom control and airflow limitation together with an immediate withdrawal of OCS therapy [32]. This quick therapeutic action probably results from the effective depletion of eosinophils induced via IL-5R blockade and ADCC-mediated apoptosis of these cells [32]. All these data together indicate that, although the improvement trend can be enhanced in the long term, asthma-control improvement is rapidly observed after at least the first three doses of benralizumab.

Patients mainly unresponsive to previous anti-IL-5 treatment and requiring stable treatment with high-dose ICS and at least one LABA were enrolled in the ORBE study. Study results suggest that benralizumab treatment could improve asthma control in patients who have previously failed to respond or discontinued treatment with mepolizumab or reslizumab. Even with its limitations, the information in this study is pertinent as it suggests that difficult to treat patients who have received, anti-IgE or anti-IL5 biologic treatment may derive benefit from being switched to bernalizumab. Recent studies and post-hoc analyses have shown that patients whose asthma did not respond to omalizumab can improve with a biologic treatment targeting the IL-5 pathway in terms of asthma control, exacerbations, and OCS reduction [30, 36–38]. Thus, benralizumab could be an effective option in severe eosinophilic asthma and could be considered as a first option before starting another biologic treatment targeting the IL-5 pathway.

The study has several limitations. It is a relatively small sample size with a short follow up period as the number of enrolled patients and length of follow-up were dictated by EAP limitations. Some additional limitations are inherent to the retrospective design such as proclivity to recall bias or misclassification bias, presence of confounding factors (other risk factors may be present that were not measured), and the inability to fully determine causation, association and temporal relationships. The absence of an active comparator arm or placebo arm makes drawing formal conclusions difficult. Due to the nature of this retrospective analysis, there is no available data of clinical information of the baseline data before initiation of any biologic treatment.

On the other hand, one of the main strengths of the ORBE study is that this is a real-life setting study describing the very first usual clinical practice with benralizumab. Besides, patients included in the study comprised a broader and more heterogeneous population than the population included in the pivotal studies. Additionally, all patients had been previously treated with other biologics. The ORBE study highlights the importance of expanded-access programmes, which allow patients with unmet clinical needs to benefit from treatment based on available evidence before health authority approval and commercial distribution.

## Conclusions

This study in a real-life setting study suggests that benralizumab is effective in in the treatment of patients with severe eosinophilic asthma and an incomplete or absent response to treatment with other biologic agents, including anti-IL5 (mepolizumab or reslizumab). Results also suggests an improvement in clinical outcomes after few doses of benralizumab indicating a rapid onset of effect, which is likely to improve over time. Benralizumab was well tolerated with a safety profile which was commensurate with previous studies.

## Data Availability

All data in the manuscript is available through the responsible corresponding author.
